# Development and comparison of a real-time PCR assay for detection of *Dichelobacter nodosus *with culturing and conventional PCR: harmonisation between three laboratories

**DOI:** 10.1186/1751-0147-54-6

**Published:** 2012-01-31

**Authors:** Sara Frosth, Jannice S Slettemeås, Hannah J Jørgensen, Øystein Angen, Anna Aspán

**Affiliations:** 1 Department of Bacteriology, National Veterinary Institute, SE-751 89 Uppsala, Sweden; 2 Norwegian Veterinary Institute, P. O. Box 750 Sentrum, N-0106 Oslo, Norway; 3 National Veterinary Institute, Technical University of Denmark, Bülowsvej 27, DK-1790 Copenhagen V, Denmark

## Abstract

**Background:**

Ovine footrot is a contagious disease with worldwide occurrence in sheep. The main causative agent is the fastidious bacterium *Dichelobacter nodosus*. In Scandinavia, footrot was first diagnosed in Sweden in 2004 and later also in Norway and Denmark. Clinical examination of sheep feet is fundamental to diagnosis of footrot, but *D. nodosu*s should also be detected to confirm the diagnosis. PCR-based detection using conventional PCR has been used at our institutes, but the method was laborious and there was a need for a faster, easier-to-interpret method. The aim of this study was to develop a TaqMan-based real-time PCR assay for detection of *D. nodosus *and to compare its performance with culturing and conventional PCR.

**Methods:**

A *D. nodosus-*specific TaqMan based real-time PCR assay targeting the 16S rRNA gene was designed. The inclusivity and exclusivity (specificity) of the assay was tested using 55 bacterial and two fungal strains. To evaluate the sensitivity and harmonisation of results between different laboratories, aliquots of a single DNA preparation were analysed at three Scandinavian laboratories. The developed real-time PCR assay was compared to culturing by analysing 126 samples, and to a conventional PCR method by analysing 224 samples. A selection of PCR-products was cloned and sequenced in order to verify that they had been identified correctly.

**Results:**

The developed assay had a detection limit of 3.9 fg of *D. nodosus *genomic DNA. This result was obtained at all three laboratories and corresponds to approximately three copies of the *D. nodosus *genome per reaction. The assay showed 100% inclusivity and 100% exclusivity for the strains tested. The real-time PCR assay found 54.8% more positive samples than by culturing and 8% more than conventional PCR.

**Conclusions:**

The developed real-time PCR assay has good specificity and sensitivity for detection of *D. nodosus*, and the results are easy to interpret. The method is less time-consuming than either culturing or conventional PCR.

## Background

Footrot is a contagious bacterial disease that affects the feet of sheep, and it has been reported in many countries [1]. The fastidious and anaerobic bacterium, *Dichelobacter nodosus*, is the main causative agent of ovine footrot [2].

In its mildest form, footrot manifests itself as a slight inflammation of the interdigital skin of sheep, but the disease may also progress to severe necrotic separation of the claw capsule from underlying tissues. Severity of the disease depends on the breed of sheep, climatic conditions, management factors and virulence of the infecting *D. nodosus *strain.

Ovine footrot was first diagnosed in Sweden in 2004 [3], and in 2008 it was detected for the first time in 60 years in Norway [4]. In 2009, the disease was diagnosed in Denmark by culture and PCR [5], however a report on clinical disease among Danish sheep was published in 1988 [6]. The emergence of ovine footrot is a challenge for the diagnostic laboratories and for the sheep industries in all three Scandinavian countries.

Clinical examination of sheep feet is fundamental to diagnosis of footrot, but detection of *D. nodosu*s should also be used to confirm the diagnosis. The presence of the typical Gram-negative rods in lesion material can be confirmed by microscopy, culturing, or PCR. Cultivation of the bacterium is time consuming and laborious, and it is an advantage to also use PCR-based detection of *D. nodosus*.

A PCR method for specific detection of *D. nodosus *was developed in 1993 by La Fontaine et al. [7], and in 2005 the method was improved by Moore et al. [8] for detection of *D. nodosus *from clinical swabs. Previously, the PCR protocol published by Moore et al. [8] was used at our institutes, but there were problems with non-specific amplicons and faint bands of the correct product size which made interpretation difficult. Moreover, conventional PCR requires agarose gel electrophoresis for identification of the PCR products, making it inconvenient for the analysis of a large number of samples. A faster and more easily interpreted method, such as real-time PCR, was desirable.

The aim of this study was to develop a TaqMan-based real-time PCR assay for detection of *D. nodosus *and to compare its performance with culturing and with conventional PCR. Another goal was to compare the sensitivity of the developed real-time PCR assay between the three laboratories participating in this study since it is advantageous to be able to use the same detection method. The real-time PCR assay was developed in collaboration between the National Veterinary Institute (SVA), the Norwegian Veterinary Institute (NVI) and the National Veterinary Institute in Denmark (DTU-VET). Its specificity (inclusivity/exclusivity) was tested at SVA and its sensitivity was tested and compared at all three laboratories. The SVA compared the real-time PCR assay with culturing for 126 Swedish sheep and the NVI compared it with conventional PCR for 224 Norwegian sheep.

## Methods

### Development of a *D. nodosus*-specific real-time PCR assay

*D. nodosus*-specific PCR primers and a TaqMan-probe targeting the 16S rRNA gene were designed using primer3 [9], producing an 84-bp fragment. The specificity of the assay was checked against GenBank sequences with the BLAST program package http://blast.ncbi.nlm.nih.gov/Blast.cgi[10]. The designed primers and TaqMan-probe were ordered from Thermo Fisher Scientific with the following sequences 5'-CGGGGTTATGTAGCTTGCTATG-3' (16Sf), 5'-TACGTTGTCCCCCACCATAA-3' (16Sr) and 5'-TGGCGGACGGGTGAGTAATATATAGGAATC-3' (16Sprobe TET-labeled). Each 20-μl PCR reaction mixture contained 1× PerfeCTa qPCR FastMix, UNG, Low Rox (Quanta BioSciences Inc., Gaithersburg, MD, USA), 0.1 mg/ml BSA (Sigma-Aldrich, St Louis, MO, USA), 0.4 μM of each primer, 0.15 μM of the TaqMan-probe and 2.5 μl of template DNA. The PCR amplification was performed in an Applied Biosystems 7500 Fast Real-Time PCR System (Applied Biosystems, Carlsbad, CA, USA), a Stratagene Mx3005P real-time PCR thermocycler (Agilent Technologies Inc., Santa Clara, CA, USA) or in a Corbett Rotor-Gene 6000 (Qiagen, Hilden, Germany) with an initial denaturation step at 95°C for 10 min, followed by 45 cycles of 95°C for 3 s and 60°C for 30 s. DNA extracted from the type strain of *D. nodosus*, CCUG 27824T (Culture Collection, University of Göteborg [CCUG], Sweden) was used as a positive control in the *D. nodosus*-specific real-time PCR and was included in each run. Every PCR run also included a non-template control in the form of DNase- and RNase-free sterile water (Sigma-Aldrich). Fluorescence signals were analysed using an automatic setting of the threshold line in the 7500 software (v.2.0.4) and a manually set threshold of 0.01 in the softwares of the Stratagene and Rotor-Gene real-time PCR instruments.

### Sensitivity of the developed real-time PCR assay and comparison of its sensitivity by three different laboratories

The detection limit and the amplification efficiency of the *D. nodosus*-specific real-time PCR assay were determined using 10-fold serial dilutions of chromosomal DNA (393 ng to 3.9 ag corresponding to approximately 3 × 10^8 ^to 3 × 10^-3 ^copies of the *D. nodosus *genome per reaction) obtained from *D. nodosus *CCUG 27824T (CCUG), and performing real-time PCR as described above. Each DNA dilution was run in triplicate in the real-time PCR analysis. The DNA from the *D. nodosus *type strain was prepared at the SVA and aliquots of the DNA preparation were sent by regular mail to the NVI and to the DTU-VET for the comparison of assay sensitivity.

### Inclusivity and exclusivity testing

Fifty-five bacterial and two fungal strains were used to test the inclusivity and the exclusivity of the developed *D. nodosus*-specific 16S real-time PCR assay (Table 1). The *D. nodosus *strains (except for strain CCUG 27824T) were obtained from the SVA strain collection whereas the strains of *Actinobacillus pleuropneumoniae, Histophilus somni *and the two *Fusobacterium*-strains were obtained from CCUG. The *Haemophilus influenzae *strain ATCC 49247 was obtained from ATCC (American Type Culture Collection). The remaining strains (*n *= 42), some of which originated from CCUG and some from the SVA strain collection, were received as DNA preparations from the Swedish Food Administration (NFA, Uppsala, Sweden) in the form of an exclusivity panel. DNA for inclusivity and exclusivity testing was extracted from colony material as described below. DNA provided by the NFA was prepared using a BioRobot EZ1 (Qiagen) and the *Fusobacterium*-DNA was prepared by a spin-column procedure (QIAamp DNA Mini Kit, Qiagen). A NanoDrop 1000 spectrophotometer (Thermo Fisher Scientific Inc., Wilmington, DE, USA) was used to measure the DNA concentrations before inclusivity and exclusivity testing. The DNA preparations were diluted in double distilled water to 2.0 ng/μl and subjected to real-time PCR analysis in duplicate, on two different occasions, as described above.

**Table 1:** Bacterial and fungal strains used for inclusivity and exclusivity testing of the developed real-time PCR assay

Organism	Strain
*Dichelobacter nodosus*	CCUG 27824T
*Dichelobacter nodosus*	AN363/05
*Dichelobacter nodosus*	AN484/05
*Dichelobacter nodosus*	07-BKT18497
*Dichelobacter nodosus*	07-BKT21558
*Dichelobacter nodosus*	07-BKT22285
*Dichelobacter nodosus*	07-BKT24952
*Dichelobacter nodosus*	08-BKT63297
*Dichelobacter nodosus*	09-BKT91977(5)
*Dichelobacter nodosus*	09-BKT94362(4)

*Actinobacillus pleuropneumoniae*	CCUG 12837
*Bacillus anthracis*	7702
*Bacillus anthracis*	4429
*Bacillus cereus*	B. cereus
*Campylobacter coli*	SLV-271
*Campylobacter jejuni*	SLV-542
*Enterobacter cloacae*	SLV-011
*Enterococcus durans*	SLV-078
*Escherichia coli*	U226
*Escherichia coli*	B266
*Escherichia coli*	L278
*Escherichia coli*	UM245
*Escherichia coli*	S262
*Escherichia coli*	XL-1 blue
*Escherichia coli EIEC*	121
*Escherichia coli O113:H21*	98NK2
*Escherichia coli O157*	SLV-479
*Escherichia coli O157*	EDL933
*Escherichia coli O157:H-*	493/89
*Escherichia coli O26:H11*	H2954/06
*Francisella tularensis*	T8
*Fusarium culmorum*	F.c
*Fusarium graminearum*	F.g
*Fusobacterium necrophorum *subsp. *funduliforme*	CCUG 42162
*Fusobacterium necrophorum *subsp. *necrophorum*	CCUG 9994
*Haemophilus influenzae*	ATCC 49247
*Histophilus somni *	CCUG 28029
*Klebsiella pneumoniae*	SLV-186
*Listeria ivanovii*	SLV-348
*Listeria monocytogenes*	SLV-513
*Proteus mirabilis*	SLV-374
*Pseudomonas aeruginosa*	SLV-395
*Pseudomonas aeruginosa*	SLV-453
*Salmonella Dublin*	SLV-242
*Salmonella Typhimurium*	SLV-248
*Shigella boydii*	33/08
*Shigella dysenterieae*	15/08
*Shigella flexneri*	100/08
*Shigella sonnei*	99/08
*Staphylococcus aureus*	SLV-438
*Staphylococcus xylosus*	SLV-283
*Vibrio cholerae*	CCUG 4070
*Vibrio parahaemolyticus*	CCUG 4224
*Vibro vulnificus*	CCUG 16397
*Yersinia enterocolitica*	SLV-408
*Yersinia pestis*	KIM
*Yersinia pseudotuberculosis*	TAVA81

### Comparison with culturing

A total of 126 Swedish sheep with clinical signs of footrot (score ≥2 foot lesions) were sampled during 2009 with the scoring system (0-5) described by Stewart and Claxton [11]. The definition of a score 2 lesion, upon which the footrot diagnosis is based in Sweden, is a necrotising inflammation of the interdigital skin involving part or all of the soft horn of the axial wall of the digit [11].

Samples were taken from the interdigital skin of the feet both for culturing and for direct real-time PCR analysis. For culturing, samples were collected using a sterile wooden stick, which was placed in Amies transport medium with charcoal (Copan Innovation Ltd, Brescia, Italy). For direct real-time PCR analysis, sample material was collected using a swab (ESwab, Copan Innovation Ltd). The samples were sent in padded envelopes by regular mail to SVA where they usually arrived within 24 hours of sampling. The samples were cultivated on hoof agar plates as described by Stewart and Claxton [11] on the day of arrival at the laboratory. The plates were incubated anaerobically at 37°C and were read after four to six days. Typical colonies were subcultured and identified on the basis of characteristic colony appearance, Gram stain and also by the real-time PCR assay specific for *D. nodosus *developed in this study.

DNA was extracted from swabs or from bacterial colonies. The swabs were first shaken for 5 min at 700-800 rpm before the fluid was transferred to a 2.0-ml microcentrifuge tube and centrifuged for 5 min at 13 000*g*. The supernatant was discarded and the pellet was resuspended in 200 μl of G2-Lysis Buffer (Qiagen). Twenty-five microliters of proteinase K (Qiagen) was added to the pellet and lysis buffer solution before lysis of the samples in a thermomixer comfort (Eppendorf, Hamburg, Germany) at 54°C and 300 rpm for 10 min. DNA extraction was performed in a BioRobot EZ1 (Qiagen) according to the manufacturer's instructions using the EZ1 Tissue Kit and the bacterial protocol from the same manufacturer. The elution volume was 100 μl and the eluate was used as template in the PCR reactions. When DNA was prepared from colony material, a loopful of bacterial colonies was picked and suspended in 450 μl of double distilled water which was incubated at 96°C for 15 min and then immediately placed on ice for at least 10 min. After centrifugation for 5 min at 13 000*g *the supernatant was used as template in the PCR reactions.

Contamination controls in the form of sterile swabs were extracted after every fifth swab processed in the DNA extraction robot. These were used as templates in the real-time PCR, in addition to a non-template control and a positive control which were included in each run.

Samples that were negative for the 16S rRNA gene of *D. nodosus *(*n *= 23) were amplified a second time with the TaqMan Exogenous Internal Positive Control Reagents (Applied Biosystems); this is an internal amplification control (IAC) for distinguishing true target negatives from PCR inhibition. The same conditions as for the 16S assay were used except that the 16S primers and probe were replaced by the control reagents.

Results from the comparison of the real-time PCR vs. culturing were plotted in a 2 × 2 table, and the agreement between results from the two methods was assessed using the kappa statistic for concordance and McNemar's test for discordance [12].

### Comparison with conventional PCR

The 224 samples for comparing the developed real-time PCR with conventional PCR were collected in Norway during 2008 and 2009. The samples were submitted to the NVI as diagnostic samples and the clinical status of the animals was unknown. A sterile wooden stick was used to collect the samples from the interdigital skin of the feet. The wooden stick was placed in a tube with sterile phosphate buffered saline (PBS) containing 0.02 M EDTA and sent to the NVI by regular mail. DNA was extracted from the samples by applying 200 μl of PBS/EDTA in a NucliSENS^® ^easyMAG^® ^extraction robot (bioMérieux, Marcy l'Etoile, France) according to the manufacturer's instructions. A prolonged lysis of ten minutes was used and the elution volume was 60 μl.

The samples were analysed using both the real-time PCR developed in this study and the conventional PCR published by Moore et al. [8] The conventional PCR method is based on amplification of a 783-bp product of the 16S rRNA gene of *D. nodosus*. Conventional PCR amplifications were carried out in an MJ Research DNA Engine DYAD^® ^PTC-0220 thermal cycler (Bio-Rad Laboratories AB, Hercules, CA, USA) with conditions described by Moore et al. [8]. Amplified DNA was run on a 1% agarose gel and visualized under UV light using a GelDoc Molecular Imager (Bio-Rad Laboratories AB).

Results from samples with high Ct values in the real-time PCR, and samples that were positive by the real-time PCR but were negative or had faint bands in the conventional PCR were cloned and sequenced for verification. These samples were run by block PCR using the real-time PCR primers, and the 84-bp PCR product was cloned using TOPO^® ^TA Cloning^® ^Kit for Sequencing (Invitrogen, Carlsbad, CA, USA) according to the manufacturer's instructions. Sequencing was performed on a capillary electrophoresis ABI-PRISM^® ^3130*xl *Genetic Analyzer (Applied Biosystems) using BigDye^® ^Terminator v1.1 Cycle Sequencing Kit (Applied Biosystems) according to the manufacturer's instructions. The 10-μl reaction mixture included 4 μl BigDye^® ^Terminator v1.1 sequencing mix, 0.5 μl primer (M13.F or M13.R), 2.5 μl ultrapurified water and 3 μl template DNA. The reaction mixture was run on a Dyad Thermal Cycler with the following conditions: denaturation at 95°C for 3 min, followed by 60 cycles containing denaturation 95°C for 45 s, annealing 50°C for 20 s and elongation 60°C for 4 min. DNA sequences were analysed using CLC Main Workbench (CLC bio, Aarhus, Denmark).

Results from the comparison of the conventional PCR vs. real-time PCR were plotted in a 2 × 2 table, and the agreement between results from the two methods was assessed using the kappa statistic for concordance and McNemar's test for discordance [12].

## Results

### Sensitivity of the developed real-time PCR assay and comparison of its sensitivity by the three different laboratories

DNA dilutions of 393 ng to 3.9 fg per PCR reaction were positive for all three replicates and were used to construct a standard curve and to determine the minimal limit of detection. The standard curve had a slope of -3.396 (R^2 ^0.998) corresponding to an amplification efficiency of 97.0% for the assay. The minimum detection limit of the developed real-time PCR assay was 3.9 fg of *D. nodosus *genomic DNA, corresponding to approximately three copies of the *D. nodosus *genome per PCR reaction. In the assay sensitivity comparison study, the same detection limit was obtained at all three laboratories.

### Inclusivity and exclusivity testing

The developed real-time PCR assay showed 100% inclusivity for the 10 *D. nodosus *strains tested and 100% exclusivity for 45 non-target bacterial strains and two non-target fungal strains (Table 1).

### Comparison with culturing

Of the 126 sheep with clinical signs of footrot (score ≥2 foot lesions), 103 (81.7%) sheep were positive for *D. nodosus *by the real-time PCR assay developed and 34 sheep (27.0%) were positive for *D. nodosus *by culturing (Table 2). The real-time PCR method found 54.8% more *D. nodosus*-positive sheep than the culturing method. The 23 samples that were negative by the *D. nodosus*-specific real-time PCR assay were all positive for the internal amplification control (IAC), which rules out negative results due to PCR inhibition.

**Table 2:** Comparison of real-time PCR and culturing

	Culturing positive	Culturing negative	Total
Real-time PCR positive	34	69	103
Real-time PCR negative	0	23	23

Total	34	92	126

A kappa value of 0.15 indicated slight agreement between the real-time PCR and culturing [12]. The relatively low value was due to the great sensitivity difference between the methods. The McNemar test value of 67 (p < 0.0001) indicates a significant difference between the two methods - the real-time PCR method detected more positive samples than did culturing.

### Comparison with conventional PCR

Results from the comparison study where 224 samples were analysed by both the developed real-time PCR and conventional PCR are presented in a 2 × 2 table (Table 3). Forty-seven (21%) of the samples had weak bands in the conventional PCR, and were sequenced for verification. Sequences were obtained for all these samples, and BLAST searches verified 34 and 13 of the samples as positive and negative for *D. nodosus*, respectively. The 13 samples that were negative for *D. nodosus *by sequencing were simultaneously negative by real-time PCR. Without the sequencing these would have represented false positive results by the conventional PCR. The 34 samples that had weak bands by conventional PCR (and that were verified as *D. nodosus *positive by sequencing) were simultaneously positive by the real-time PCR.

**Table 3:** Comparison of real-time PCR and conventional PCR (C-PCR)

	C-PCR positive	C-PCR negative	Total
Real-time PCR positive	71^a^	17	88
Real-time PCR negative	0	136^b^	136

Total	71	153	224

^a^34 of the samples run by C-PCR were sequenced; ^b^13 of the samples run by C-PCR were sequenced.

Hence, taking results from sequencing into consideration, there were differences between real-time PCR and conventional PCR in 17 of the 224 samples. In all of these 17 cases, real-time PCR was positive and conventional PCR was negative, and the real-time PCR products were successfully cloned and sequenced. A BLAST search verified all the 17 samples as *D. nodosus *with a 100% score http://blast.ncbi.nlm.nih.gov/Blast.cgi.

A kappa value of 0.84 indicated good agreement between the two methods [12], but the McNemar test value was 15.1 (p < 0.0001) indicating a significant difference between the two methods - the real-time PCR method detected more positive samples than the conventional PCR.

## Discussion

Good diagnostic tools are essential to identify the presence of *D. nodosus *and to study its epidemiology. Such knowledge is important to limit and control footrot, a disease that constitutes a major animal welfare problem. A correct diagnosis is a prerequisite to distinguish footrot from other diseases or conditions that can affect the feet of sheep such as contagious ovine digital dermatitis (CODD), white line disease, granulomas and toe and pedal joint abscesses [13].

Footrot is often introduced into a sheep flock by the purchase of an infected animal and transmission within a flock occurs mainly from sheep to sheep via the environment [14]. The environment can also be a source for introduction of footrot as reported by Whittington et al. [15] where a flock became infected after using the same yard used by an infected flock some hours earlier. The risk of introduction or re-introduction of footrot into a flock can be reduced by some principal preventive strategies as described by Abbott and Lewis [16]: animals should only be purchased from footrot-free flocks, purchased animals should be quarantined, or the flock should be sequestered from outside introduction.

In this study, a TaqMan-based real-time PCR assay targeting the 16S rRNA gene for the detection of *D. nodosus *in clinical samples was developed in collaboration between SVA, NVI and DTU-VET. The approach was chosen with the aim of improving detection of *D. nodosus *compared to traditional culturing and conventional PCR. The real-time PCR assay was compared to culturing for 126 Swedish sheep by the SVA and to conventional PCR for 224 Norwegian sheep by the NVI. Its specificity (inclusivity/exclusivity) was tested at SVA and its sensitivity was tested and compared at all three laboratories. It is an advantage that the same detection method with the same sensitivity can be used in the three different Scandinavian countries, so that results can be easily compared.

Another real-time PCR targeting *D. nodosus *has recently been published by Calvo-Bado et al. [17] but its emphasis is on quantification rather than detection. This assay is based on the *rpoD *gene which is a single copy gene in the *D. nodosus *genome while the developed real-time PCR in this study is based on the 16S rRNA gene which exists in three copies [18]; this is an advantage when sensitive detection is required.

There was a significant difference between the real-time PCR assay and culturing with the real-time PCR method being three times more sensitive in detecting positive samples. This is not surprising because *D. nodosus *is a fastidious organism that can be difficult to culture, particularly when samples are not plated immediately. It was, however, somewhat surprising that the total number of real-time PCR positive samples was not higher (81.7%) as all samples were from sheep with clinical signs of footrot (score ≥2 foot lesions). One explanation could be that sampling, which took place in the field and by different persons, was not optimal and that sample quality deteriorated in the post. In a footrot prevalence study by König et.al. [19], in which sampling took place at the laboratory and by the same persons, 97% of the samples from sheep with score 2 footrot lesions were found positive with the same real-time PCR developed in this study and 79% by culturing.

When weak bands in the conventional PCR were sequenced there was good agreement between conventional PCR and the developed real-time PCR method. However, the real-time PCR method detected 8% more positive samples compared to the conventional PCR.

In the 13 samples found to be negative by the real-time PCR, the conventional PCR gave a faint band of approximately the correct size. Without sequencing, these samples could have been incorrectly interpreted as *D. nodosus *positive. At the NVI this was a severe problem when *D. nodosus *diagnostics were first implemented there. Of approximately 6000 samples analysed by the conventional PCR in a screening study in 2008 [20], 11% had to be sequenced due to diffuse bands. Of the sequenced samples, 75% of these were found not to be *D. nodosus *after a BLAST search (Jannice S Slettemeås, personal communication).

A main advantage with the probe-based, real-time PCR over conventional PCR is that it eliminates non-specific amplicons and faint bands of the correct product size. A great proportion (21%) of the conventional PCR products had to be sequenced. The real-time PCR is more sensitive, less time consuming and laborious, and does not involve post-PCR processing. A greater sensitivity of real-time PCR compared to conventional PCR has also been shown in previous studies [21] and [22]. Real-time PCR is a good tool for identifying slow-growing bacteria like *D. nodosus*. Probe-based real-time PCR provides specificity, i.e. it limits some of the nonspecific fluorescence signals toward the end of the reaction [23].

There were no signs of inhibition in the developed real-time PCR assay in this study, but inhibitors can vary with sample material. Inhibition is also dependent on the DNA purification method used, so one should run an IAC simultaneously with the samples or in a subsequent assay.

## Conclusions

The developed real-time PCR assay is a specific and easy-to-interpret method for detection of *D. nodosus*, and it is more sensitive and faster than either culturing or conventional PCR. There is an advantage that the same detection method is used in the three Scandinavian countries, so that results can be easily compared. A rapid and reliable detection method will aid in diagnosis and efforts to reduce the incidence of, or even to eradicate, virulent footrot in sheep populations. The developed real-time PCR is, however, not intended as a replacement for culturing as isolation of *D. nodosus *is still required for virulence testing and fingerprinting. However, it is a good complement to the laborious conventional culturing techniques.

Future studies could include determining the detection limit of *D. nodosus *in artificially contaminated swab samples. In addition, an IAC could be incorporated in the real-time PCR assay instead of running it separately as today.

## Competing interests

The authors declare that they have no competing interests.

## Authors' contributions

The study was designed by SF and AA. SF, JSS and ØA performed the laboratory work and the analysis of results was done by all authors. HJJ performed all statistical calculations. SF drafted the manuscript and all authors revised, read and approved the final manuscript.
